# Global patterns of the cranial form of modern human populations described by analysis of a 3D surface homologous model

**DOI:** 10.1038/s41598-022-15883-3

**Published:** 2022-08-15

**Authors:** Hirofumi Matsumura, Toyohisa Tanijiri, Makiko Kouchi, Tsunehiko Hanihara, Martin Friess, Vyacheslav Moiseyev, Chris Stringer, Kengo Miyahara

**Affiliations:** 1 School of Health Sciences, Sapporo Medical University, Sapporo, 060-8556 Japan; 2 Medic Engineering, Kyoto, 606-8181 Japan; 3 National Institute of Advanced Industrial Science and Technology, Tokyo, 135-0064 Japan; 4 Department of Anatomy, Kitasato University, Sagamihara, 252-0373 Japan; 5 Département Homme et Environnement, Musée de l’Homme, 75116 Paris, France; 6 Peter the Great Museum of Anthropology and Ethnography (Kunstkamera), Russian Academy of Sciences, St Petersburg, 199034 Russia; 7 Department of Earth Sciences, The Natural History Museum, London, SW7 5BD UK; 8 Kyoto City Archeological Research Institute, Kyoto, 602-8435 Japan

**Keywords:** Anthropology, Biological anthropology, Anatomy, Skeleton

## Abstract

This study assessed the regional diversity of the human cranial form by using geometric homologous models based on scanned data from 148 ethnic groups worldwide. This method adopted a template-fitting technique for a nonrigid transformation via the iterative closest point algorithm to generate the homologous meshes. Through the application of principal component analysis to 342 sampled homologous models, the largest variation was detected in overall size, and small South Asian crania were clearly verified. The next greatest diversity was found in the length/breadth proportion of the neurocranium, which showed the contrast between the elongated crania of Africans and the globular crania of Northeast Asians. Notably, this component was slightly correlated with the facial profile. Well-known facial features, such as the forward projection of the cheek among Northeast Asians and compaction of the European maxilla, were reconfirmed. These facial variations were highly correlated with the calvarial outline, particularly the degree of frontal and occipital inclines. An allometric pattern was detected in facial proportions in relation to overall cranial size; in larger crania, the facial profiles tend to be longer and narrower, as demonstrated among many American natives and Northeast Asians. Although our study did not include data on environmental variables that are likely to affect cranial morphology, such as climate or dietary conditions, the large datasets of homologous cranial models will be usefully available for seeking various attributions to phenotypic skeletal characteristics.

## Introduction

Geographic variations in the human cranial form have been studied for a long time. Numerous researchers have assessed the diversity in regards to environmental adaptation and/or natural selection, particularly in terms of climatic factors^1–7^ or mastication functions related to dietary conditions^5,8–13^. Furthermore, some studies focused on the bottleneck effect, genetic drift, gene flow, or random evolutionary processes caused by neutral genetic mutation^14–23^. For instance, the spherical shape of the broader and shorter cranial vault is attributed to adaptation with selective pressure to an extremely cold climate by Allen’s rule^24^, which postulated that mammals minimize heat loss by reducing body surface area against volume^2,4,16,17,25^. In addition, some studies adopting Bergman’s rule^26^ explained cranial size in relation to temperature^3,5,16,25,27^, suggesting that the total body size in cold regions tended to be greater to protect against heat loss. The mechanical effects of masticatory stress on the growth patterns of the cranial vault and facial skeleton have been argued^8,9,11,12,28^ in light of dietary conditions caused by differences in cooking culture or subsistence between agriculturalists and hunter-gatherers. The general interpretation is that a reduction of mastication stress diminishes the robustness of the facial skeleton and muscles. Some global studies have attributed the diversity of cranial shape patterns primarily to the phenotypic outcomes of neutral genetic distance instead of environmental adaptation^21,29–32^. Another explanation for changes in cranial shape is based on the concepts of isometry or allometry^6,33–35^. For instance, the evolutionary process through which bigger brains tended to possess relatively wider frontal lobes at the so-called Broca’s cap region with increasing frontal breadth was addressed on the basis of allometry^34^. In addition, a study investigating secular changes in cranial shape detected an allometric trend of brachycephalization (a trend toward globular crania) with an increase in stature^33^.

The long history of research on cranial morphology includes attempts to identify the primary factors of various aspects that contribute to the diversity in cranial shape. The traditional methods used in many of early studies were based on 2D linear measurement data, often with Martin’s or Howell’s definitions^36,37^. Meanwhile, many of the studies mentioned above adopted more advanced methods based on the spatial 3D geometric morphometrics (GM) technique^5,7,10–13,17,20,27,34,35,38,39^. For example, the sliding semi-landmark approach based on minimizing bending energy has been the most commonly used method in GM biology. It projects the semi-landmarks of the template to every specimen by sliding along curves or surfaces^38,40–46^. Including such superimposition techniques, most 3D GM studies use Generalized Procrustes Analysis, which is referred to as the iterative closest point (ICP) algorithm^47^, thus making the shapes directly comparable and thereby capturing variation. Alternatively, the thin plate spline (TPS) technique^48,49^ has also been widely used as a nonrigid transformation method for matching semilandmark alignment with a shape on a grid base.

With the development of practical 3D whole-body scanners, sizing surveys using 3D body scanners have been conducted in many studies since the end of the twentieth century^50,51^. The scanned data have been used to extract body dimensions, which required the surface shape to be described as a surface rather than a point cloud. Template fitting is a method developed for this purpose in the field of computer graphics, in which the surface shape is described by the polygon mesh model. The first step in template fitting is the preparation of a mesh model that will serve as a template. Some vertices that constitute the template are landmarks. Then, the template is deformed and fitted to the surface to minimize the distance between the template and point cloud while preserving the local shape features of the template as much as possible. The landmarks in the template match those in the point cloud. Using template fitting, all scan data can be described as mesh models with the same number of data points of the same topology. Although the exact homology is only present in the landmark positions, an overall homology between the created models can be assumed because the changes in the geometric structure of the template are minimal. Therefore, mesh models created by template fitting are sometimes called homologous models^52^. The advantage of template fitting is that the template can be deformed and fitted to different parts of a target object that are spatially close but far from the surface (such as the zygomatic arch and temporal region of the skull) without affecting each other’s deformations. Therefore, the template can be fitted to a branched object, such as a trunk with the upper arms in a standing posture or the hand. The disadvantage of template fitting is the high computing cost of repeated iterations; however, as a result of significant improvements in computer performance, this is no longer a problem. By analyzing the coordinate values of the vertices that make up the mesh models using a multivariate analysis method, such as principal component analysis (PCA), variations in the entire surface shape can be analyzed, and virtual shapes at arbitrary positions in the distribution can be calculated and visualized^53^. Nowadays, mesh models created using template fitting have been widely used for shape analyses in various fields^52,54–60^.

Advancements in non-rigid mesh registration technique, together with the rapid development of portable 3D scanning devices capable of scanning at higher resolutions, speeds, and mobility than CT, have facilitated the recording of 3D surface data regardless of location. Thus, in the field of bioanthropology, such novel technologies have enhanced the potential for quantitative evaluation and statistical analysis of human specimens, including cranial specimens, which are the focus of the present study.

**Figure 1:**
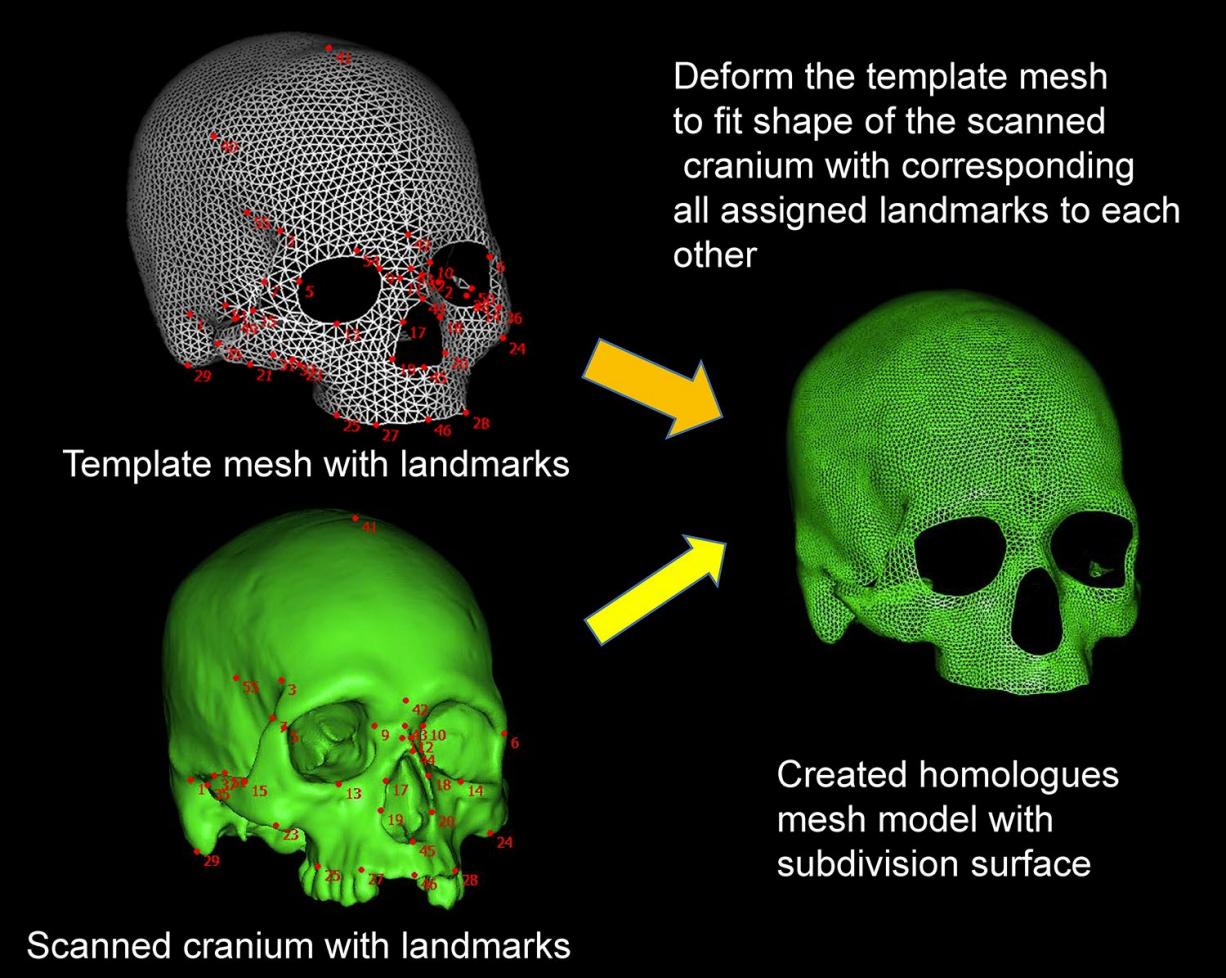
Creation process of the homologous fitting model of 3D scanned crania.

Therefore, using such advanced 3D homologous modeling technique based on template-fitting (Fig. 1), this study assessed the diversity of cranial forms by performing geographic comparisons on a global scale on the basis of 342 cranial specimens selected from 148 populations worldwide (Table 1). To interpret the variations in cranial form, we applied PCA and receiver operating characteristic (ROC) analysis to the datasets of our created homologous models. The results will provide a better understanding of global variations in cranial form, including regional patterns and the descending order of variations, correlative variations between cranial segments, and the existence of allometric trends. Although this study does not deal with the data for external variables represented by climate or dietary conditions that likely affect cranial form, the geographical patterns of cranial form recorded in our study will be useful for exploring ecological, biomechanical, and genetic factors of cranial variation.

**Table 1:**
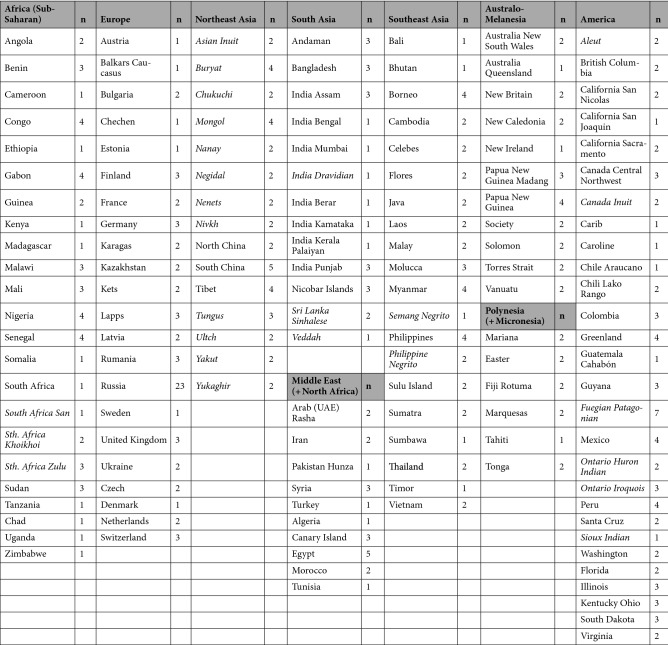
Cranial specimens (males) used for this study from 9 geographical units in the world (Italic:ethnic group).

## Results

### PCA and ROC analysis

Table 2 presents the eigenvalues and contribution rate of PCA applied to the non-normalized datasets of 17,709 vertices (53,127 XYZ coordinates) of the 342 homologous cranial models. Consequently, 14 primary principal components were detected with contribution rates greater than 1% of the total variance, with the cumulative proportion of variances being 83.68%. The loading vectors of the 14 principal components are recorded in Supplementary Table S1, and component scores computed for the 342 cranial samples are provided in Supplementary Table S2.

**Table 2:** Results of PCA adopted to the coordinates data sets of 342 homologous cranial models.

Component	Eigenvalue	Contribution rate (%)	Cumulative rate (%)
1	14,627.0	27.53	27.53
2	6688.8	12.59	40.12
3	5331.4	10.04	50.16
4	4099.1	7.72	57.87
5	3277.2	6.17	64.04
6	2984.8	5.62	69.66
7	1819.1	3.42	73.09
8	1219.0	2.30	75.38
9	1070.4	2.02	77.39
10	868.7	1.64	79.03
11	707.3	1.33	80.36
12	607.8	1.14	81.50
13	600.4	1.13	82.63
14	553.6	1.04	83.68

**Figure 2:**
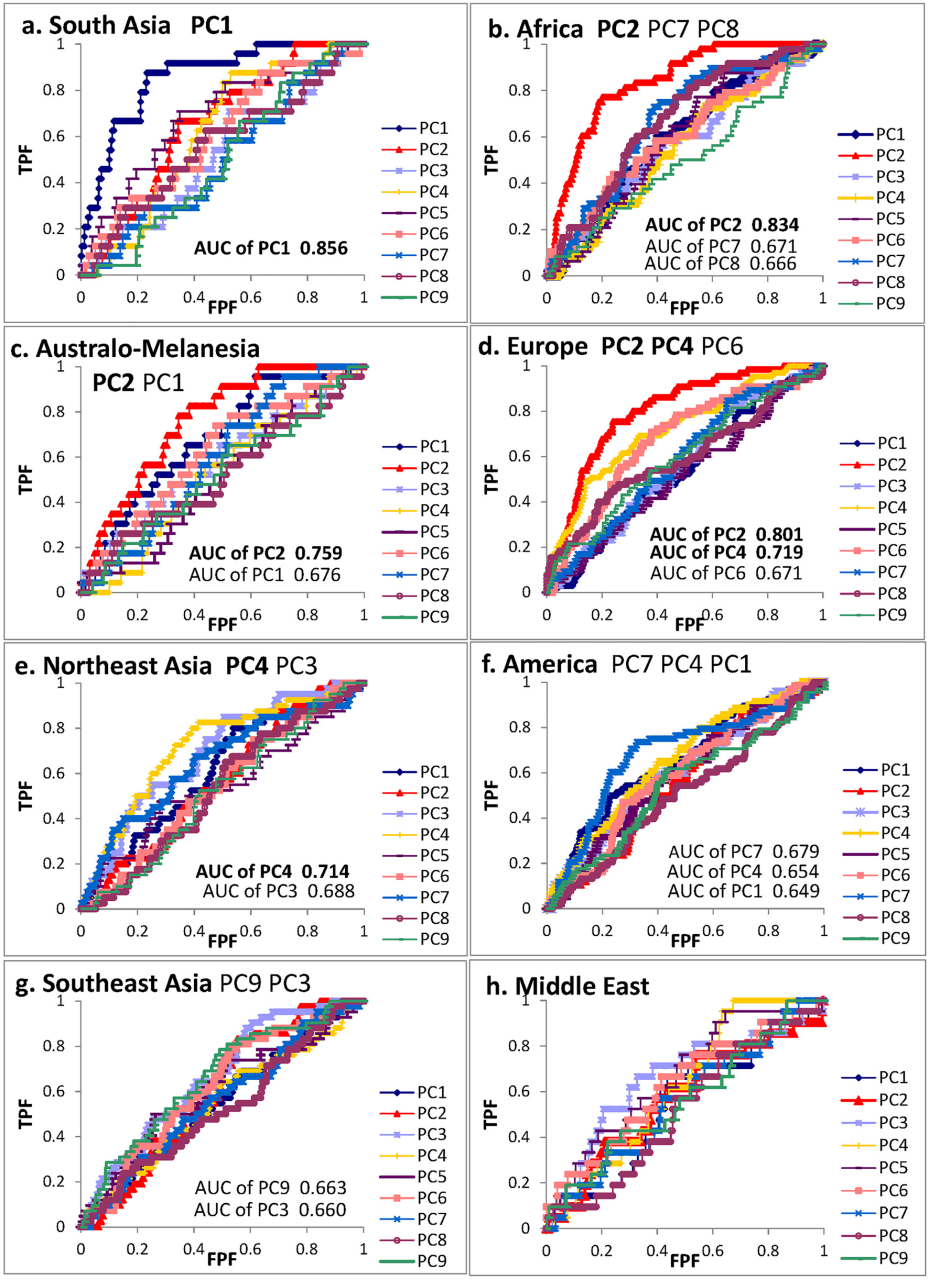
ROC curves applied to nine principal component scores based on vertex data sets of 342 male homologous cranial models. AUC: area under the curve with significance at 0.01% level to distinguish each geographical assemblage from other pooled assemblage. *TPF* true-positive fraction (effective discrimination), *FPF* false-positive fraction (non-effective discrimination).

This study evaluated the first nine components with contribution rates greater than 2%, some of which showed substantial and significant geographical diversities in the cranial form. Figure 2 depicts the curves drawn by ROC analysis to elucidate the most effective component of the PCA to characterize or divide each sample assemblage by major geographic units (e.g., division between African and non-African). The Polynesian assemblage was not tested because of the small sample size used for this test. The significance data for differences in AUC and other basic statistics computed by ROC analysis are shown in Supplementary Table S3.

The interpretations of ROC curves are summarized below, focusing only on components through which the comparative group sets were distinguishable with considerably or relatively large AUCs and high significance levels with probability under 0.001. The South Asian assemblage (Fig. 2a), mainly consisting of samples from India, was clearly distinguished from other geographically pooled samples by a prominently larger AUC (0.856) for the first component (PC1) in comparison with those for other components. The African assemblage (Fig. 2b) was specifically characterized by a comparably large AUC (0.834) for PC2. Australo-Melanesians (Fig. 2c) showed a similar tendency to Sub-Saharan Africans by PC2 with a relatively large AUC (0.759). Europeans (Fig. 2d) were clearly distinguished by a combination of PC2 (AUC = 0.801), PC4 (AUC = 0.719), and PC6 (AUC = 0.671); Northeast Asian specimens (Fig. 2e) were considerably distinguished by PC4, with a relatively large AUC of 0.714, and faintly by PC3 (AUC = 0.688). The following groups were also discriminated with lower AUC values and a high significance level: the findings for PC7 (AUC = 0.679), PC4 (AUC = 0.654) and PC1 (AUC = 0.649) indicated that American natives (Fig. 2f) possess specific features correlating to these components, Southeast Asians (Fig. 2g) were distinguished by PC3 (AUC = 0.660) and PC9 (AUC = 0.663), but the patterns of specimens from the Middle East (Fig. 2h), including North Africa, were not highly distinctive from others.

**Figure 3:**
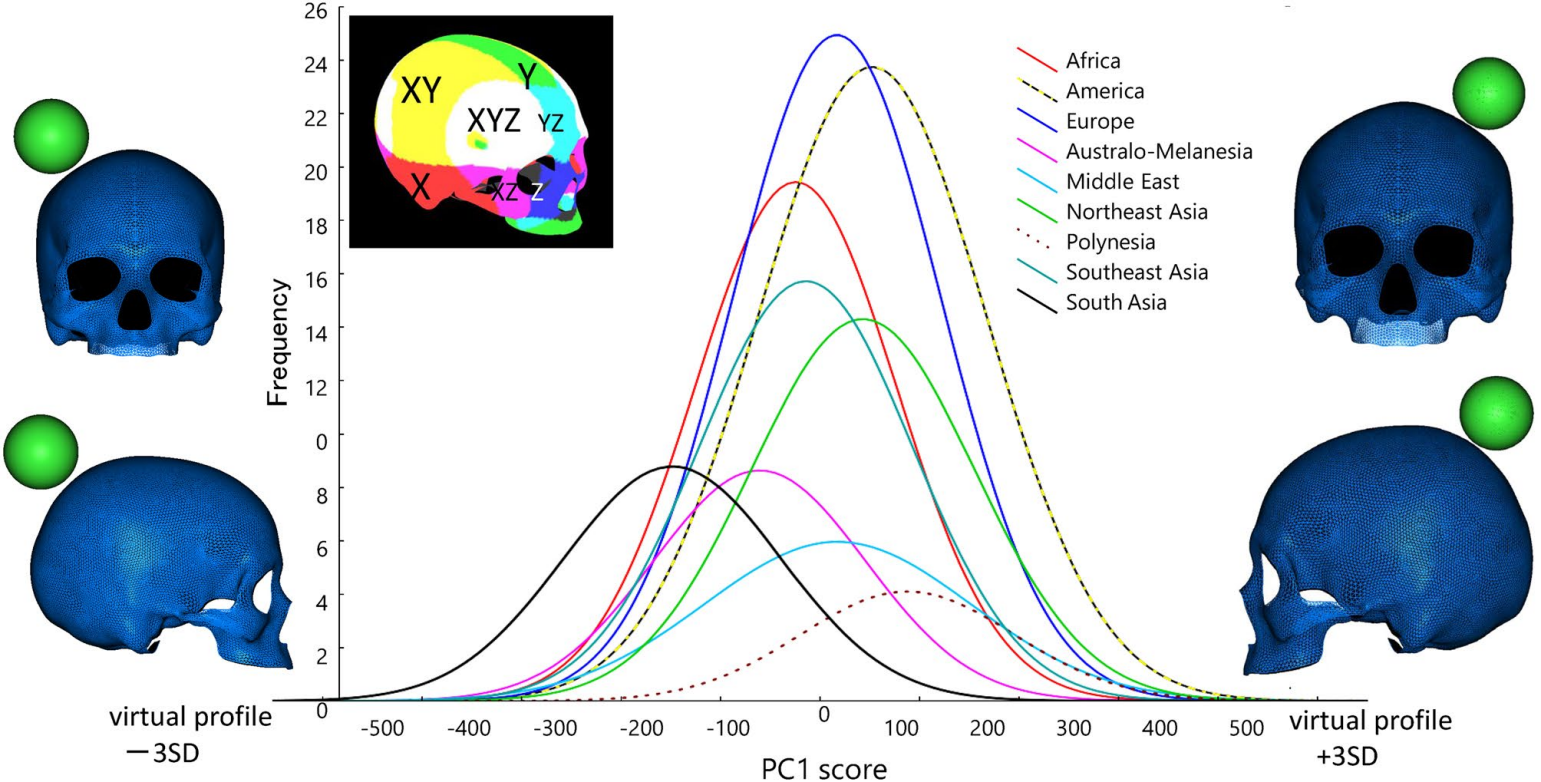
Frequency distribution (normal fit curve) for PC1 scores of 342 male cranial individuals worldwide, a color map of cranial surface highly correlating vertices to PC1 (interpretation of color against X, Y, Z axis: see text), and virtual shape deformations with a magnitude of 3 SD at the opposite sides of this axis. The scale is given as a green colored sphere of 50 mm in diameter.

In the next step, to visually interpret highly correlating vertices, surface zones with high loading values greater than 0.45 were colored with X, Y, and Z coordinate information, as shown in Fig. 3. The red zone shows a high correlation with the coordinates of the X-axis corresponding to the horizontal transverse direction. The green zone is highly correlated with the coordinates of the perpendicular Y-axis, and the dark blue area is highly correlated with the Z-coordinate axis in the sagittal direction. The light blue zone is correlated to both the Y- and Z-coordinate axes; pink is the mixed zone correlated to the X- and Z-coordinate axes; yellow represents the zone correlated to the X- and Y-coordinate axes; and the white zone is reflected by X-, Y-, and Z-coordinate axes. Consequently, at this loading value threshold, PC 1 is mostly correlated with the entire cranial surface. The virtual cranial forms with a magnitude of 3 SD at the opposite sides of this component axis are simultaneously depicted in this figure, and the morphing image is provided in Supplementary Video S1, visually confirming that PC1 contains the factor of overall cranial size.

Figure 3 shows the frequency distribution graphs (normal fit curve) of individual PC1 scores calculated separately for nine geographical units. In addition to being estimated by ROC curves (Fig. 2), the scores of South Asians are distinctively plotted toward the left side to a certain extent because their crania are smaller than those of the other regional groups. As listed in Table 1, these South Asians represent ethnic groups in India, including the Andaman and Nicobar Islands, Sri Lanka, and Bangladesh.

**Figure 4:**
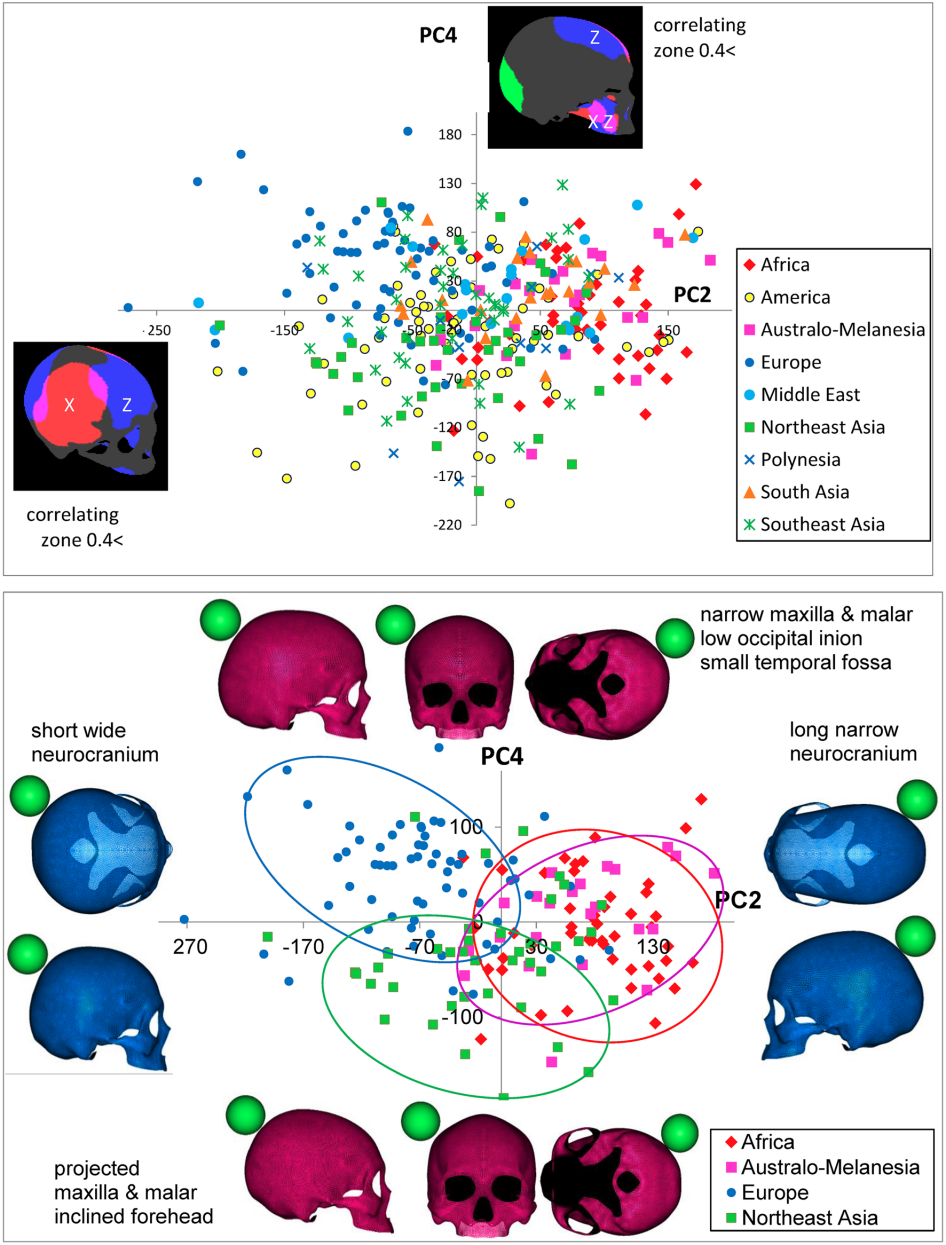
Scatter scoring diagrams of PC2 and PC4 for cranial individuals from nine geographical units (upper), and four geographical units (lower), color maps of cranial surface highly correlating vertices to each PC (interpretation of color against X, Y, Z axis: see text), and virtual shape deformations with a magnitude of 3 SD at the opposite sides of these axes. The scale is given as a green colored sphere of 50 mm in diameter.

**Figure 5:**
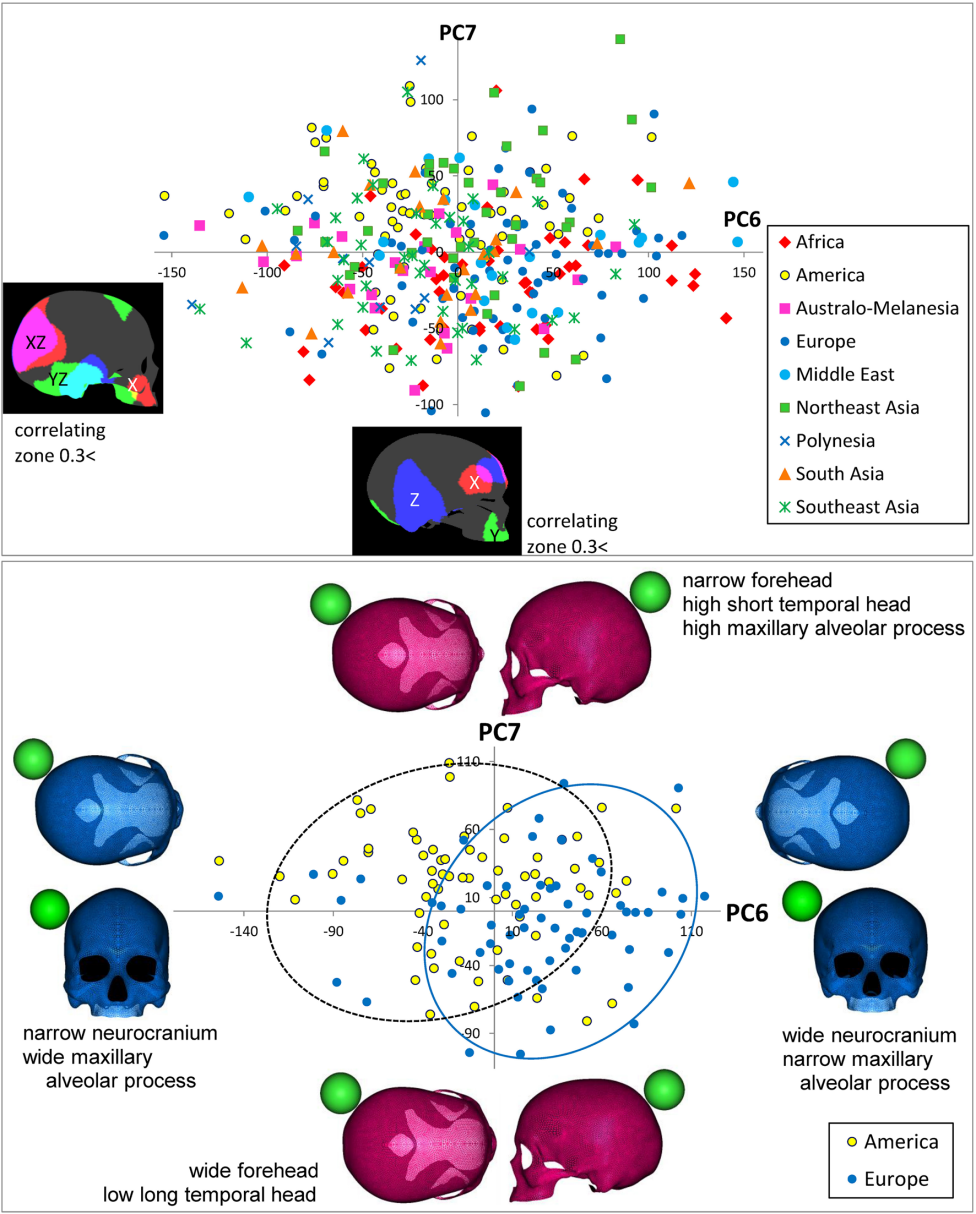
Scatter scoring diagrams of PC6 and PC7 for cranial individuals from nine geographical units (upper), and two geographical units (lower), color maps of cranial surface highly correlating vertices to each PC (interpretation of color against X, Y, Z axis: see text), and virtual shape deformations with a magnitude of 3 SD at the opposite sides of these axes. The scale is given as a green colored sphere of 50 mm in diameter.

**Figure 6:**
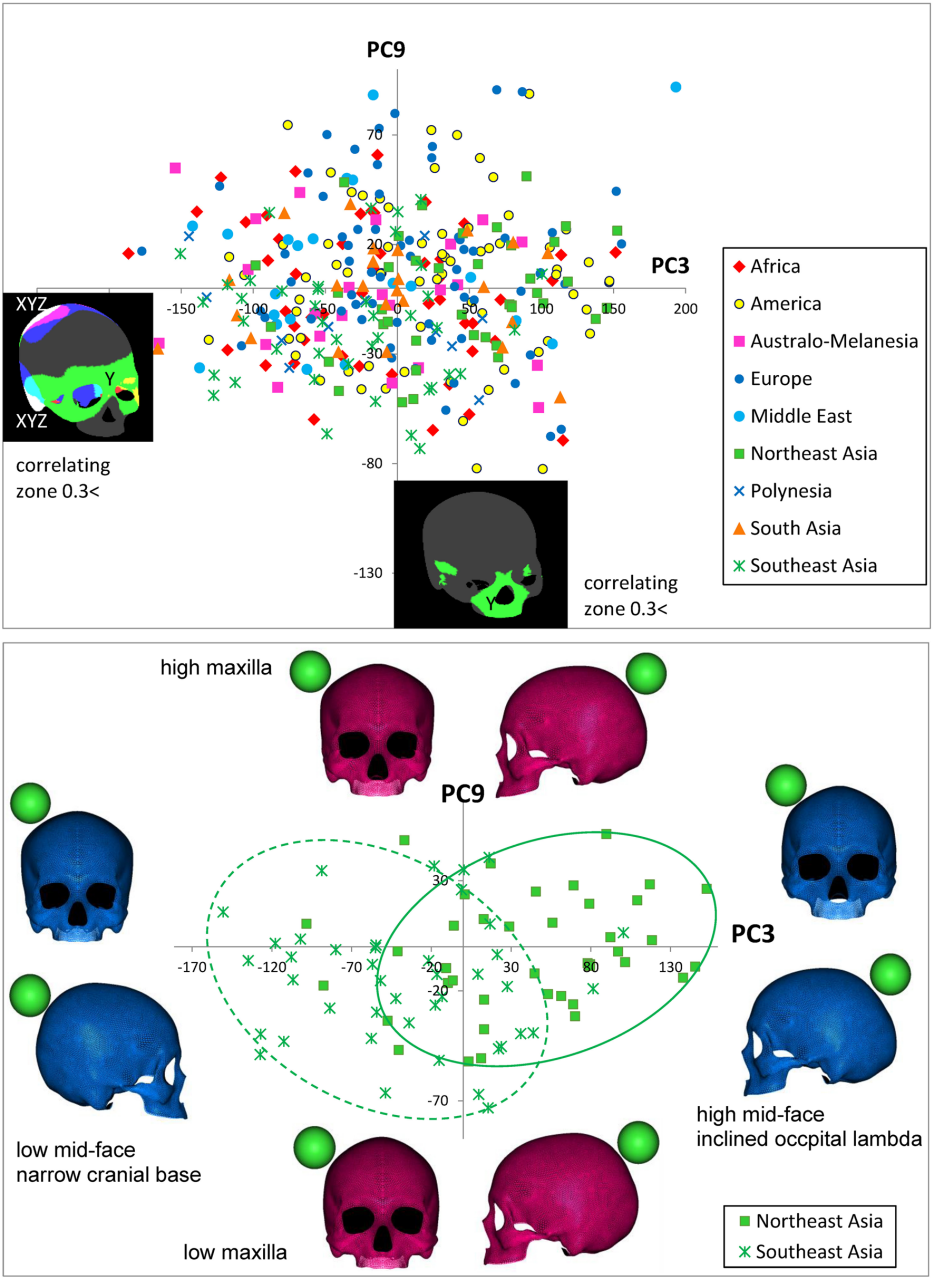
Scatter scoring diagrams of PC3 and PC9 for cranial individuals from nine geographical units (upper), and three geographical units (lower), color maps of cranial surface highly correlating vertices to each PC (interpretation of color against X, Y, Z axis: see text), and virtual shape deformations with a magnitude of 3 SD at the opposite sides of these axes. The scale is given as a green colored sphere of 50 mm in diameter.

The size factor was detected in PC1. As a consequence of seeking highly correlating zones and virtual shapes, the shape factors were elucidated as components other than PC1; although, the size factor was not always removed completely. As described by ROC curve comparisons (Fig. 2), PC2 and PC4 were the most discriminative, followed by PC6 and PC7. PC3 and PC9 were fairly effective in dividing the sample assemblages by geographical units. Accordingly, scatter diagrams of PC scores and colored surfaces highly correlated to each component and virtual shape deformations with a magnitude of 3 SD at the opposite sides are schematized in these pairs of component axes (Figs. 4, 5, 6). The convex hulls covering samples of each geographical unit drawn in these figures are approximately 90%, although the clusters overlap to a certain extent. Table 3 summarizes the interpretations of each PCA component.

**Table 3:** Interpretations of distinguishable components in PCA between homologous cranial models by geographical units.

Principal component	Interpretation	geographic units effectively distinguished through ROC analysis (/ = versus)
PC1	Overall cranial size	South Asia/others
PC2	Length/breadth proportion of neurocranium	Africa, Australo-Melanesia/Europe
PC3	Mid-facial height inclination of occipital lambda breadth of cranial base	Northeast Asia/Southeast Asia
PC4	Cheek breadth and projection space of temporal fossa inclination of frontal bone position of occipital inion	Northeast Asia, America/Europe
PC5	Relative size of the mastoid processes of the temporal bone	*ns*
PC6	Inclination of alveoli height/breadth proportion of neurocranium	Europe/others
PC7	Forehead breadth temporal head width height of alveolar process	America/others
PC8	Asymmetry of the overall cranial shape	*ns*
PC9	Maxillary height	Southeast Asia/others

*ns* no significant.

In the chart plotting PC2 and PC4 scores (Fig. 4, Supplementary Videos S2, S3 show the morphing images), surface color maps were simultaneously exhibited when the threshold of the loading value was set over 0.4, which is a lower level than in PC1 because the overall loading values are smaller in PC2 than in PC1.

The frontal and occipital elongations toward the sagittal direction along the Z-axis (dark blue) and the parietal surface toward the X-axis along the coronal direction (red) were highly correlated with PC2, whereas PC4 was correlated with both the Z- and X-axes (pink) of the cheek bone and the Y-axis (green) of the occipital part and the Z-axis (dark blue) of the frontal part. In this chart, the scores of all individuals used worldwide were plotted; however when all samples consisting of a large number of groups were plotted together at once, the interpretation of the scattering pattern was quite complex due to the substantial overlap; therefore, samples from only four major geographical units, namely, African, Australo-Melanesian, European, and Northeast Asian, are scattered below this chart with virtual cranial deformations with a magnitude of 3 SD in this PC scoring range at the opposite sides. In this figure, with a scoring pair of PC2 and PC4, Africans much overlapping with Australo-Melanesians were plotted on the right side, while Europeans were scattered on the left upper area, and Northeast Asians tended to be assembled toward the left lower side. The horizontal axis of PC2 demonstrated African/Australo-Melanesians possessing relatively longer neurocrania than the others. PC4, in which Europeans and Northeast Asian assemblages were loosely separated, is linked to the relative size and projection of cheek bones, together with the lateral outline of the cranial vault. The scoring patterns demonstrate that Europeans have a relatively narrow maxilla and malar bones, smaller space of temporal fossa surrounded by a zygomatic arch, and a perpendicularly elevated frontal and flat occipital bone with a lower inion, while Northeast Asians tend to have wide protruding cheek bones with an inclined frontal and rising occipital base.

When focusing on PC6 and PC7 (Fig. 5) (Supplementary Videos S4, S5 show the morphing images), the coloring chart, with the threshold of loading value over 0.3, indicates that PC6 is related to the maxillary or alveolar form (red: X and green Y: axis), shape of temporal bone (light blue: Y and Z axis), and occipital form (pink: X axis and Z axis). PC7 correlates with the maxillary anterior alveolar height (green: Y-axis) and the form of the head side around parieto-temporal part along the Z-axis (dark blue), in addition to the forehead width (red: X-axis). In the upper diagram of Fig. 5, all geographical samples are scattered based on the component scores of PC6 and PC7. Since the ROC suggested that PC6 consists of European-specific features, and PC7 characterizes American natives in this analysis, these two regional samples were selectively plotted in this pair of component axes. American natives, despite being sampled from a broad area, are scattered on the upper left side; conversely, many European samples tended to be plotted on the right lower side. The pair of PC6 and PC7 characterizes the Europeans’ narrow alveolar process and relatively wide neurocranium, whereas American natives are characterized by a narrower forehead and larger maxilla with a wide high alveolar process.

ROC analysis suggested that PC3 and/or PC9 characterize Southeast Asians and Northeast Asians. Correspondingly, the scoring pair of PC3 (green-colored upper-face segment on the Y-axis) and PC9 (green-colored lower-face segment on the Y-axis) (Fig. 6; Supplementary Videos S6, S7 provide the morphing images) schematizes the diversity of East Asians, in contrast to the high facial proportion of Northeast Asians and the low facial shape of Southeast Asians. In addition to these facial features, the inclination of the occipital bone at the lambda portion was detected as another characteristic of some Northeast Asians, while a narrow cranial base was described for a part of Southeast Asians.

The above description of the principal components and descriptions of PC5 and PC8 were skipped because specific regional features were undetected among the nine major geographic units. PC5 is related to the size of the mastoid processes of the temporal bone, and PC8 reflects the asymmetry of the overall cranial shape, both of which show parallel variations among the nine geographic sample assemblages.

In addition to the scatter diagram of the PCA scores at the individual level, we provided scatter plots by group averages for gross comparisons. For this purpose, average cranial homologous models were generated based on vertex datasets of individual homologous models of 148 ethnic groups. The two-dimensional plots of the score sets of PC2 and PC4, PC6 and PC7, and PC3 and PC9 are depicted in Supplementary Fig. S1, all of which were calculated as average cranial models of 148 population samples. The scatter diagram thus hides individual variation within each population, enabling more clear interpretation of cranial affinities due to major regional distributions, in which the patterns correspond to those depicted by individual plotting, with less overlap. The grand-average models by each geographical unit are exhibited in Supplementary Fig. S2.

### Allometry

Except for PC1, which relates to overall size (Supplementary Table S2), the allometric relationships between the overall cranial size and form were explored using centroid size and PCA score sets derived from non-normalized data. The allometric coefficients, constant values, t-values, and P-values in the significance tests are presented in Table 4. At the level of P < 0.05, no significant allometric patterns relating to overall cranial size were detected in any of the cranial form components.

**Table 4:** Allometry between centroid size and PCA scores with contribution rate greater than 1.0%.

Principal component	Results using PCA scores based on non-normalized data sets				Results using PCA scores based on normalized data sets			
	Allometric coefficient	Constant	t-value	P-value	Allometric coefficient	Constant	t-value	P-value
PC1					− 1.1142	16.0582	− 1.4655	0.1437
PC2	1.3440	− 6.9244	1.8216	0.0694	− 1.3750	18.1652	− 1.5235	0.1286
PC3	− 0.2616	7.5698	− 0.2513	0.8017	− 0.2748	8.1528	− 0.4116	0.6809
PC4	− 0.5856	10.6655	− 0.6526	0.5145	0.6243	− 0.3936	0.8994	0.3691
PC5	0.3331	2.3526	0.5958	0.5517	− 1.0960	− 15.3920	− 1.3767	0.1695
PC6	− 0.6945	11.4155	− 0.6706	0.5030	3.0287	− 23.4644	2.6984	0.0073**
PC7	− 0.1325	5.7981	− 0.1224	0.9027	0.1810	− 23.4644	0.2645	0.7915
PC8	0.1656	3.2827	0.2450	0.8066	0.6299	− 1.0205	0.8627	0.3889
PC9	0.2617	1.9004	0.2467	0.8053	− 2.4312	−26.7118	− 1.8482	0.0655
PC10	− 0.0217	4.5250	− 0.0260	0.9792	− 1.9361	−22.4834	− 2.3452	0.0196*
PC11	− 0.2766	6.8866	− 0.3419	0.7326	1.1896	− 6.4948	1.6783	0.0942
PC12	− 0.4622	8.5476	− 0.5868	0.5577	1.6759	− 11.1435	1.8068	0.0717
PC13	0.0385	4.0534	0.5439	0.5869	1.1292	− 5.8749	1.9123	0.0567
PC14	− 0.3288	7.2899	− 0.3986	0.6905	− 0.3289	7.1296	− 0.3247	0.7456
PC15					1.0131	− 5.4731	0.8078	0.4198

*Significance at 5% level.

**1% level.

**Figure 7:**
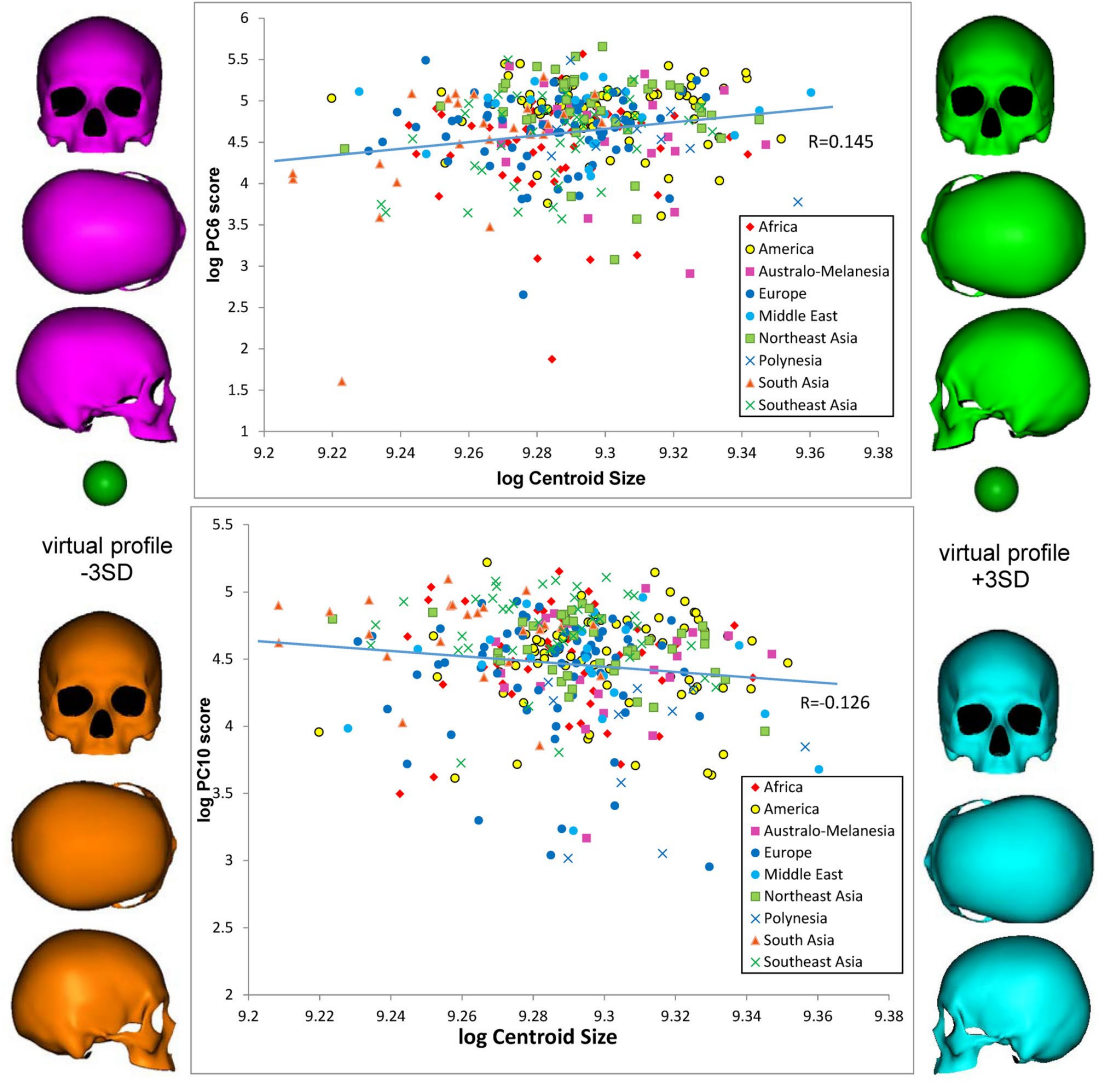
The regression slopes in log liner relations between the PC scores of shape components (derived from normalized data) and centroid sizes, with virtual shape deformations having a magnitude of 3 SD at the opposite sides of the lines, for the significant allometric relationships listed in Table 4.

Because there is a possibility of some size factor being included in the PC scores based on non-normalized data sets, we further investigated allometric trends between centroid size and PC scores computed using data sets normalized by centroid size (the PCA results and score sets are provided in Supplementary Tables S6, S7). The results of this allometric analysis are presented in Table 4. Consequently, significant allometric trends were detected at the 1% level in PC6 and at 5% level in PC10. Figure 7 shows regression slopes in log liner relations between these PC scores and centroid sizes, with virtual shapes at both ends of the log centroid size (± 3 SD). The PC6 scores are indicative of the relative cranial height and breadth in proportion. As cranial size increases, the cranial vault and the face become higher, while the forehead and the orbital and nasal openings tend to be transversely tight. The scattering pattern of the specimens demonstrates that Northeast Asians and American natives tend to have this proportion. In addition, PC10 shows a tendency for proportionally narrower midfacial breadth, regardless of the geographical unit.

## Discussion

Through analysis of datasets of homologous 3D surface models, the following patterns of variation were demonstrated in the cranial form. The first component in PCA was related to the overall size of the cranium. The small crania of South Asians, including specimens from India, Sri Lanka, and Bangladesh Andaman, have long been known to be associated with their small body size corresponding to Bergmann’s Rule or Island Rule^61^ based on ecogeographical conditions^3,5,16,25,27,62^. The former is related to temperature, and the latter depends on the available space in the niche and food resources. In the shape components, the largest variation was detected in the length/breadth ratio of the cranial vault. This characteristic was assigned PC2, which describes the close affinity between the proportionally elongated crania of Australo-Melanesians and Africans and dissimilarities with the globular crania of some Europeans and Northeast Asians. These characteristics have been reported in many previous studies based on data from simple linear measurements^37,63,64^. In addition, this feature is related to brachycephalization in non-Africans, which has long been argued in somatometric and osteometric studies. The major hypothesis underlying this interpretation is that masticate reduction, such as gracilization of the temporal muscles, reduces stress at the lateral side of the head^5,8–13^. Another hypothesis is linked to adaptation to cold climates by reducing the surface area of the head, in accordance with Allen’s rule^16,17,25^, assuming that a more globular cranial vault minimizes the surface area more than a spheroid shape. Based on the findings of the current study, these hypotheses are assessable only on the basis of inter-correlation of cranial segments. Consequently, our PCA results do not completely support the hypothesis that the cranial length/breadth ratio is substantially affected by masticating conditions, since the loadings of PC2 (long/short head component) are not considerably correlated with the facial proportion, including relative maxillary size and relative space of the temporal fossa (reflecting volume of temporal muscle). Our current study did not analyze the relationship between cranial shape and geo-environmental conditions such as temperature; nonetheless, interpretations based on Allen’s rule might be worth considering as a candidate hypothesis to explain the short-rounded crania in cold climate regions.

Next, remarkable variation was detected in PC4, characterizing Northeast Asians as possessing large protruding cheek bones over the maxillary and zygomatic bones. This finding corresponds to a specific feature well-known among Siberians, who have been thought to adapt to an extremely cold climate through increased maxillary sinus volume and flat face due to a forward-positioned zygomatic bone^65^. A novel finding obtained with our homologous model is that the posterior setback cheeks of Europeans are linked to a reduction in the frontal incline and flat narrow occipital bone with lower inion. In contrast, northeast Asians tend to possess an inclined forehead with a lifted occipital region. An investigation^35^ of the occipital bone using geometric morphometric techniques revealed that Asian and European crania are characterized by flatter occipital curvature and lower inion positions in comparison with Africans. However, our scatter diagrams for the PC2 and PC4 and PC3 and PC9 pairs exhibited great variation among Asians, whereas in Europeans, the characteristics of a flat occipital base with a lower inion. The discordance in Asian features between the studies was probably due to differences in the ethnic samples used, and we used samples from a large number of ethnic groups from a broad region encompassing Northeast and Southeast Asia. The variations in occipital shape have generally been associated with muscle development. However, such an adaptive interpretation does not account for the correlation between forehead and occipital shape, which has been demonstrated in this study and has been scarcely well-argued. In this regard, the relationship between weight balance and the center of gravity or the point of the cervical junction (foramen magnum), or else factor may be worth considering.

Another major component with great diversity is related to the development of masticating organs represented by the maxilla and temporal fossa, as described by the scoring combination of PC6, PC7, and PC4. A considerable reduction in these cranial segments characterizes European samples better than any other geographical group. This feature has been interpreted as a reduction in the robustness of facial morphology due to early development of agriculture and cooking style, which in turn reduces mechanical stress on the masticatory organs without a strong chewing apparatus^9,12,28,66^. According to the “masticatory-functional” hypothesis^28^ this is accompanied by a change in flexion of the cranial base to a more acute basicranial angle and a more globular cranial vault. From this perspective, agricultural populations tend to possess compact faces without prognathic jaws and with more globular brain cases. This deformation can thus be interpreted as an overall outline of the cranial lateral shape of Europeans with reduced masticating organs. However, based on this study, this interpretation is difficult because the functional significance of morphological conjunctions between the globular neurocranium and the development of mastication organs is less acceptable, as addressed in the former interpretation of PC2.

The difference between Northeast Asians and Southeast Asians is represented by the contrast of the high face with the inclined occipital lambda and low face with the narrow cranial base, as demonstrated in PC3 and PC9. Our study provides only a limited explanation for this finding owing to the lack of geo-ecological data. Adaptation to different climates or dietary conditions is a possible explanation. In addition to environmental adaptation, local differences in population history between Northeast and Southeast Asia were considered. For instance, in East Eurasia, a two-layer model was hypothesized to understand the dispersal of anatomically modern humans (AMH) based on cranial morphometric data^67,68^. According to this model, the “first layer”—that is, the original Late Pleistocene AMH colonizing population—shares a more or less direct ancestry with initial occupants of this region akin to current Australo-Melanesians (first layer), later undergoing large admixture with agricultural people from the north who possess Northeast Asian features (second layer) into this region (ca. 4000 BP). This gene flow drawn by the “two-layer” model will be required to understand the cranial shape of Southeast Asians, considering the possibility that Southeast Asians’ cranial shape was affected by genetic inheritance from indigenous first layers to a certain extent.

By evaluating cranial affinities using a geographical unit drawn by the homologous model, we can suggest the major population history of AMH through the out-of-Africa scenario. A number of different “out of Africa” models have been proposed to explain AMH dispersal based on skeletal and genomic data. Of these, most recent studies have documented that the colonization of regions outside Africa by AMH dates back to approximately 177,000 years ago^69,70^. However, the remote dispersal of AMH over Eurasia during this period is still uncertain because these earlier fossil localities were restricted to the Middle East and the Mediterranean region near Africa. The simplest scenario suggests a single dispersal from Africa to Eurasia along migration routes, avoiding geographic barriers such as the Himalayan mountains^71–74^. Another model proposes multiple waves of migration, with the first dispersal from Africa occurring along the Indian Ocean Rim into Southeast Asia and Australia, followed by subsequent dispersal to northern Eurasia^20,22,64,74–77^. Most of these studies accept an early stage of AMH dispersal to a region distant from Africa approximately 60,000 years ago. In this regard, Australo-Melanesian (including Papuan) samples exhibited a closer resemblance to African samples than any other geographic series in the PCA of the homologous model. This finding supports the hypothesis that the first dispersal group of AMH along the southern rim of Eurasia was of direct African origin^22,68^, without significant morphological alterations in accordance with a particular climate or other substantial conditions.

Concerning allometry, analysis using shape components derived from another data sets normalized by centroid size demonstrated significant allometric trends in PC6 and PC10. Both components relate to the shape over the forehead and facial segments, profiling a proportionally narrower forehead and face as the cranial size increases. The relatively large crania of Northeast Asians and American natives tend to possess this characteristic. This finding contradicts a previously reported allometric pattern in which larger brains possessed relatively wider frontal lobes in the so-called Broca cap region, resulting in an increase in the frontal breadth^34^. The discrepancy is attributed to the difference in sample sets; our study analyzed an allometric pattern in overall cranial size using a modern population, while the contrasting study addressed long-term trends in human evolution relating to brain size.

In terms of facial allometry, a study using biometric data^78^ documented that facial shape and size may not be significantly related, while our study demonstrated that a larger cranium tends to be associated with a high narrower face. However, the consistency of the biometric data is unclear; regression tests comparing ontogenetic allometry with static allometry showed different results. An allometric trend toward a globular cranium due to an increase in stature^33^ has also been reported; however, we did not analyze stature data. Our study did suggest that there was no allometric data for an association between cranial globular proportions and the overall cranial size itself.

Although our current study did not deal with data of external variables represented by climate or dietary conditions, which are highly likely to affect cranial morphology, the large datasets of homologous 3D cranial surface models used in this study will be useful in assessing phenotypic morphological variation associated with environmental factors, such as diet, climate, and dietary conditions, and neutral forces such as migration, gene flow, and genetic drift.

## Methods

### Cranial specimens and 3D surface scanning

This study included 342 male cranial specimens selected from 148 populations from nine geographic units (Table 1). Most of the groups were geographically native specimens, whereas some groups (listed in italics) in Africa, Northeast/Southeast Asia, and America were defined by ethnicity. Many of cranial specimens were selected from cranial measurements database after Martin’s craniometric definitions^79^ provided by Tsunehiko Hanihara. We chose representative male skulls from each ethnic group worldwide. To identify the individuals representing each population, we calculated the Euclidean distance based on 37 cranial measurements from the population mean to all individuals belonging to the population. In most cases, we selected 1–4 specimens with the smallest distance from the mean (Supplementary Table S4). For the groups, some specimens were randomly selected if they were absent from the list in Hanihara’s measurement database.

For statistical comparisons, 148 population samples were grouped according to the major geographical units, as shown in Table 1. The group “Africa” consisted only of samples from the sub-Saharan regions. The samples from North Africa were included in the “Middle East,” together with the environmentally similar West Asians. The “Northeast Asia” group included only people of non-European origin, and the “America” group consisted only of Native Americans. In particular, this group is distributed over a broad area of the North and South American continents in different environments. Regardless, we dealt with American samples in this single geographical unit, considering the population history of Native Americans who are assumed to be of Northeast Asian origin, regardless of multiple migrations^80^.

We recorded the 3D surface data of these comparative cranial specimens by using a high-resolution 3D scanner (EinScan Pro produced by Shining 3D Co Ltd, minimum resolution: 0.5 mm, https://www.shining3d.com/) and then generated mesh wire models consisting of approximately 200,000–400,000 vertices, using the included software to fill holes and smooth edges.

### Generation of the homologous model

#### Template with landmark setting

In the first step, we created a single-template mesh model of the cranium consisting of 4485 vertices (8728 polygon faces) using scan data from an arbitrary cranium. From the template mesh model, the area of the cranial base consisting of the sphenoid bone, petrous part of the temporal bone, palate, maxillary alveolar, and teeth were removed. The reason was that these structures are sometimes incomplete or difficult to complete due to thin or fine sharp segments such as pterygoid planes and styloid process, tooth abrasion, and/or inconsistency of tooth sets. The cranial base around the foramen magnum, including the basion point, was not removed because it is an anatomically important position with which the cervical vertebrae are articulated, and it is necessary to assess cranial height. The template model was formed bilaterally symmetrically by using a mirror ring. Isotropic remeshing was performed to transform the polygon shape to be as equilateral as possible.

Next, 56 landmarks were assigned using the HBM-Rugle software to the anatomically corresponding vertices of the template model. Landmark setting generates accuracy and stability for landmark localization and ensures homology in these locations in the created homologous models. They are identifiable according to their specific characteristics, as shown in Supplementary Table S5 and Supplementary Fig. S3. Most of these landmarks were type I landmarks at the intersection of three structures, according to Bookstein’s definitions^81^, and some others are of type II with maxima of curvature points. Many landmark points were diverted from the points in Martin’s definition^36^ that were determined for linear cranial measurements. We defined the same 56 landmarks that were manually allocated on the anatomically corresponding vertices for the scanned models of 342 cranial specimens for the purpose of generating more accurate homologous models in the next section.

#### Head-oriented coordinate system

A head-oriented coordinate system was defined to describe the scan data and template as shown in Supplementary Fig. S4. The X–Z plane is the Frankfurt horizontal plane that passes through the highest point on the upper margin of the right and left external auditory canals (Martin’s definition: porion) and the lowest point on the lower margin of the left orbit (Martin’s definition: orbitale). The X axis is the line connecting the right and left porions, and X+ is the right porion. The Y–Z plane passes the midpoint of the right and left porions and the nasion; Y+ is upward and Z+ is forward. The reference point (origin: zero coordinate) was set at the intersection of the Y–Z plane (median plane), X–Z plane (Frankfort plane), and X–Y plane (coronal plane).

#### Template fitting

We used HBM-Rugle software (Medic Engineering, Kyoto, http://www.rugle.co.jp/) to create homologous mesh models by template fitting using 56 landmarks (left side of Fig. 1). The main components of this software were developed originally at the Digital Human Research Center, National Institute of Advanced Industrial Science and Technology, as HBM, which has functions for template fitting using landmarks and creating fine-mesh models using subdivided surfaces^82^. In the later version of this software (mHBM)^83^, a function for template fitting without using landmarks was added, and the fitting performance was improved. HBM-Rugle incorporates mHBM software and has additional functions for user convenience, including setting coordinate systems and size adjustment of input data. The robustness of the fitting accuracy of this software has been verified in several studies^52,54–60^.

In HBM-Rugle template fitting using landmarks, the template mesh model is imposed onto the target scan data by rigid registration based on the ICP technique (minimizing the sum of distances between the corresponding landmarks of the template and target scan data); the template is then fitted onto the target scan data by non-rigid mesh deformation. This fitting process was repeated three times using different values for two of the fitting parameters to improve the fitting accuracy. One of these parameters penalizes for the distance between a template mesh model and target scan data, and the other parameter penalizes the distance between template landmarks and target landmarks. The deformed template mesh model was then subdivided by the Loop subdivision surface algorithm^82^ to create a finer mesh model consisting of 17,709 vertices (34,928 polygons). Lastly, the subdivided template mesh model was fitted to the target scan data to create a homologous model. Since the landmark positions are slightly different from those of the target scan data, the homologous models were fine-tuned so that they were described using the head-oriented coordinate system described in the previous section. The mean distance between the corresponding landmarks of the homologous model and the target scan data was < 0.01 mm in all samples. The mean distance between the data points of the homologous model and the target scan data was 0.322 mm, as calculated using a function of HBM-Rugle (Supplementary Table S2).

### PCA and ROC analysis

To interpret the variations in cranial form, the 17,709 vertices (53127 XYZ coordinates) of all homologous models were analyzed by principal component analysis (PCA) using the software HBS produced by Digital Human Research Center at the National Institute of Advanced Industrial Science and Technology, Japan (distributer: Medic Engineering, Kyoto, http://www.rugle.co.jp/). Then, we attempted to apply PCA to the non-normalized dataset and the dataset normalized by centroid size. Consequently, PCA based on non-normalized data resulted in clearer characterization of the cranial form by the nine geographical units, and easier interpretation of components, as compared with PCA using normalized data.

This paper presents the number of primary principal components that were detected with a contribution rate greater than 1% of the total variance. To identify the principal component(s) that are most effective in differentiating groups by major geographical units, a receiver operating characteristic (ROC) analysis^84^ was applied to the principal component (PC) scores with the contribution rate greater than 2%. This analysis generates a probability curve for each PCA component to improve the classification performance and correct plotting between the comparative geographical groups. The degree of capability of distinguishing is assessable by the area under the curve (AUC), of which the component of PCA with greater value is at better distinguishing between groups. Then, the chi-square test was performed to evaluate the significance level. ROC analysis was performed in Microsoft Excel using the Bell Curve for Excel (version 3.21) software.

To visualize geographic differences in cranial form, scatter diagrams were created using the PC scores that most effectively differentiated groups from major geographical units. To interpret the principal components, the vertices of the model that were highly correlated with a principal component were visualized using a color map. In addition, virtual representations at both ends of a principal component axis located at ± 3 standard deviations (SD) of the principal component scores were calculated and are provided in Supplementary Videos.

### Allometry

Allometry was used to define the relationship between the cranial shape and size factors estimated in the PCA analysis. The analysis was applied to the primary components with contribution rates > 1%. One limitation of this PCA is that the form components might be not be indicative of shape alone because the non-normalized data sets did not have all size factors removed. In addition to using non-normalized data sets, we analyzed allometric trends using PC score sets based on data normalized by centroid size, applied to the primary components with contribution rates > 1%.

The allometric trend^85^ was tested using the equation Y = aX^b^, where Y is the score of the form or shape component, X is the centroid size (Supplementary Table S2), a is a constant value, and b is the allometric coefficient. This method primarily introduced allometric studies in geometric morphometrics^78,86^. The logarithmic transformation of this formula is log Y = b × log X + log a. For computations of a and b, regression analysis using the least-squares method was applied. When performing a logarithmic transformation for Y (centroid size) and X (PC score), these values must be positive; nonetheless, the score set of X includes negative values. As a solution, we added a rounded the absolute value of the minimum score plus 1 to each score in each component, and logarithmic transformation was applied to all converted positive scores. The significance of the allometric coefficients was assessed using a two-tailed Student’s t-test. These statistical calculations to test allometry were performed using the Bell Curve for Excel software (version 3.21).

## Supplementary Information

Supplementary Figure S1.

Supplementary Figure S2.

Supplementary Figure S3.

Supplementary Figure S4.

Supplementary Table S1.

Supplementary Table S2.

Supplementary Table S3.

Supplementary Table S4.

Supplementary Table S5.

Supplementary Table S6.

Supplementary Table S7.

Supplementary Video S1.

Supplementary Video S2.

Supplementary Video S3.

Supplementary Video S4.

Supplementary Video S5.

Supplementary Video S6.

Supplementary Video S7.

Additional Information.
