# Supplementary figures and images for: Global patterns of the cranial form of modern human populations described by analysis of a 3D surface homologous model
Source: Sci Rep. 2022 Aug 15;12:13826. doi: 10.1038/s41598-022-15883-3 (PMC9378707; doi:10.1038/s41598-022-15883-3)

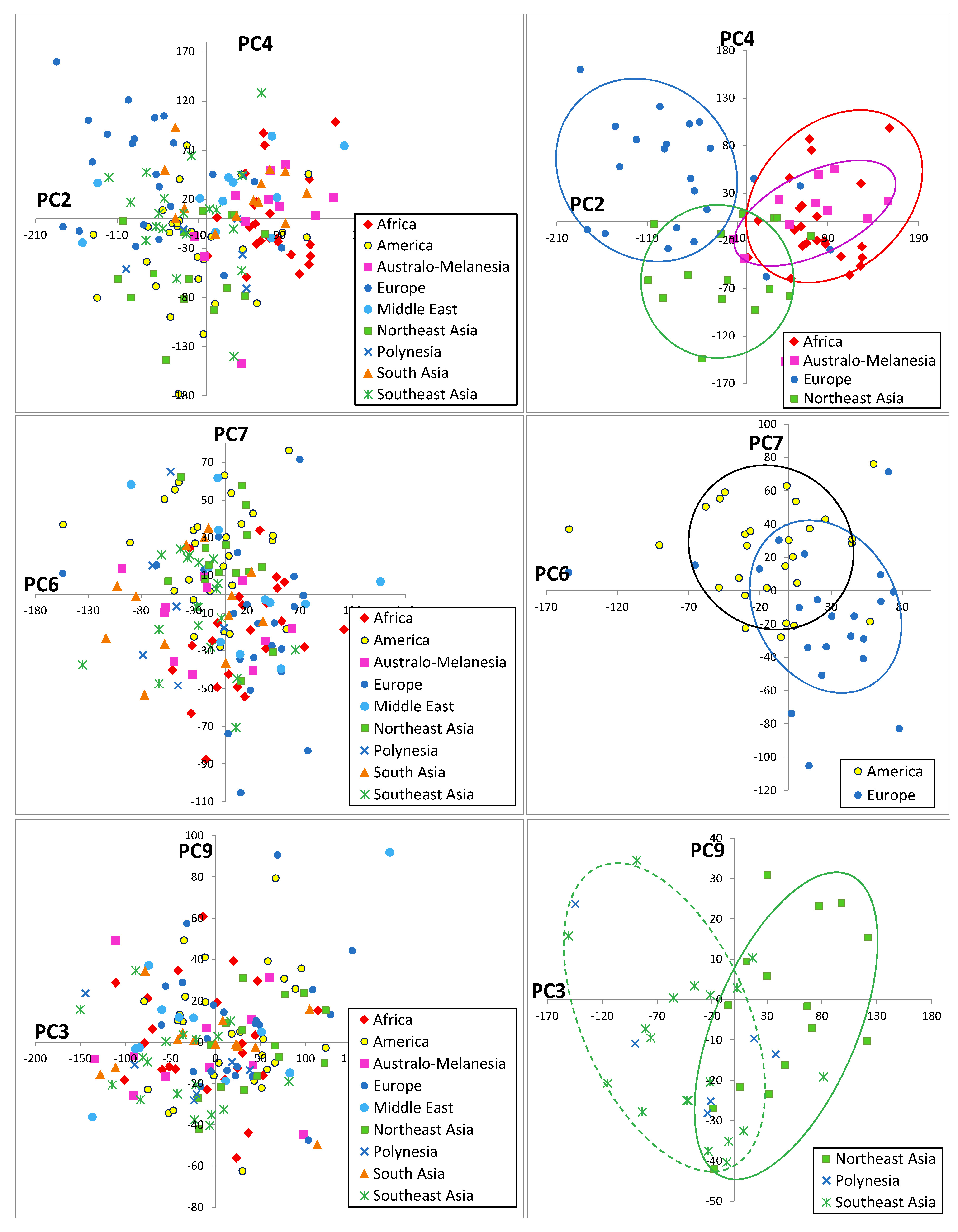

Supplement: Supplementary file 1 — Supplementary Figure S1. [file 41598_2022_15883_MOESM1_ESM.jpg]

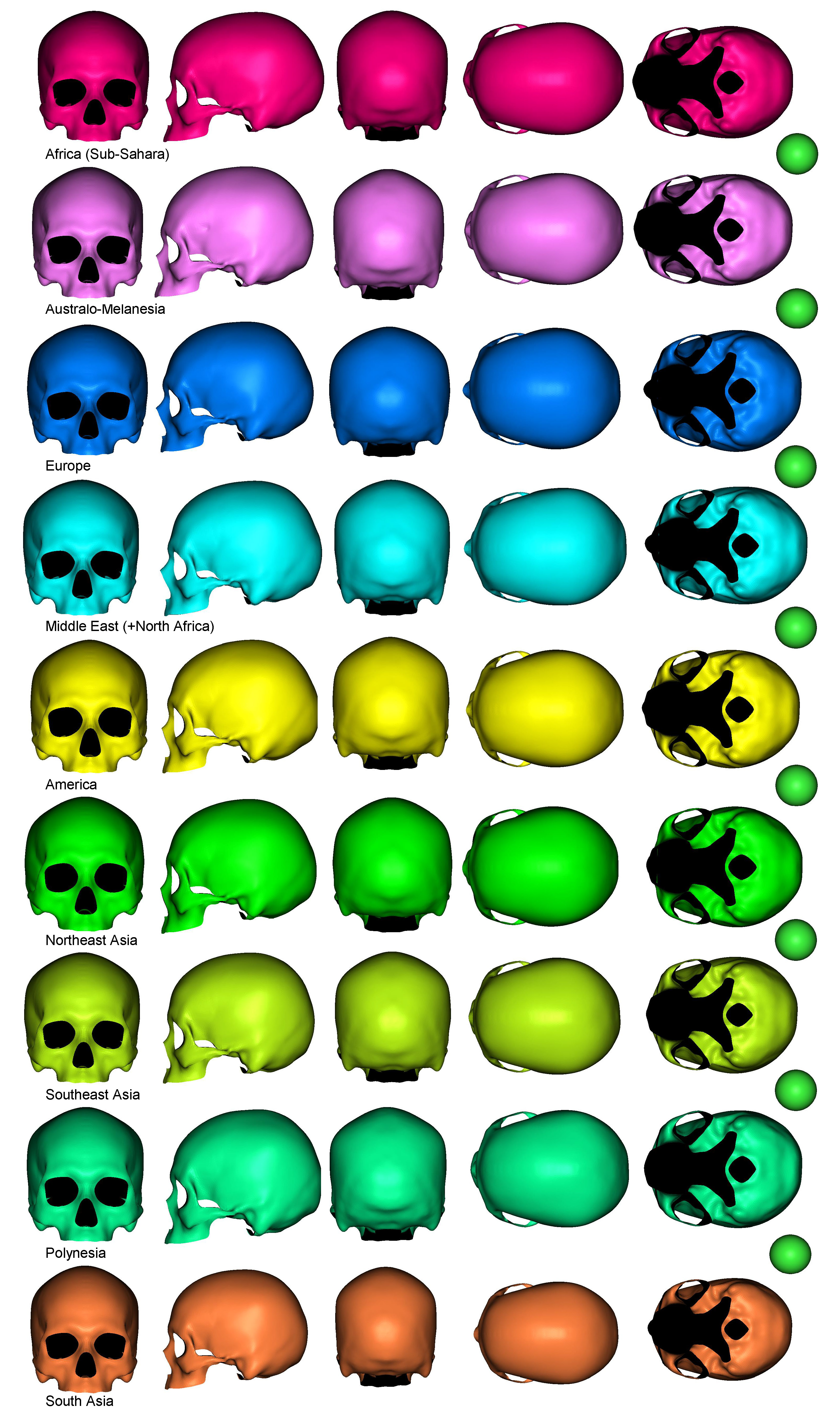

Supplement: Supplementary file 2 — Supplementary Figure S2. [file 41598_2022_15883_MOESM2_ESM.jpg]

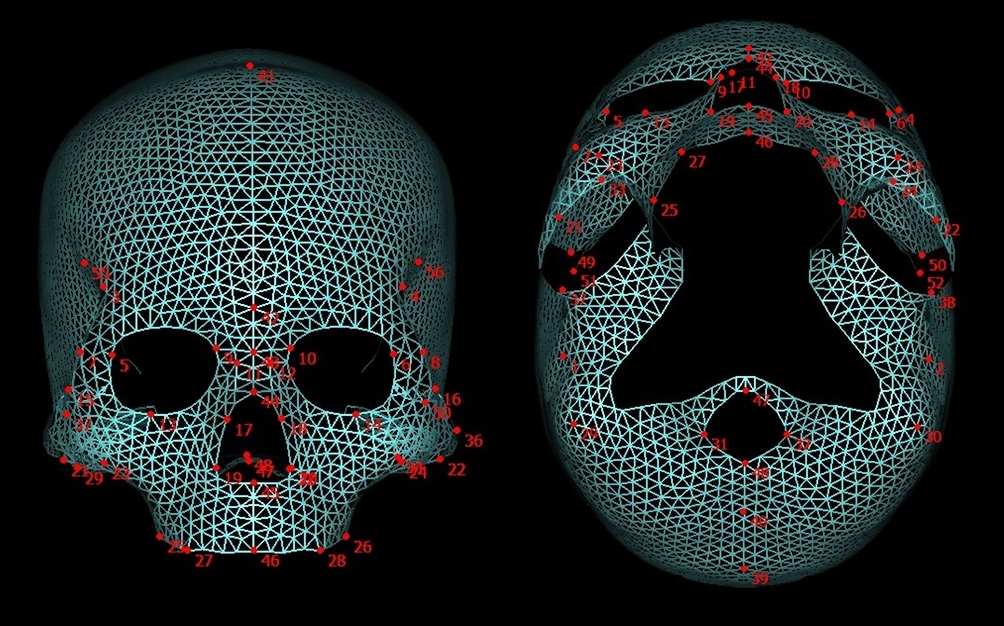

Supplement: Supplementary file 3 — Supplementary Figure S3. [file 41598_2022_15883_MOESM3_ESM.jpg]

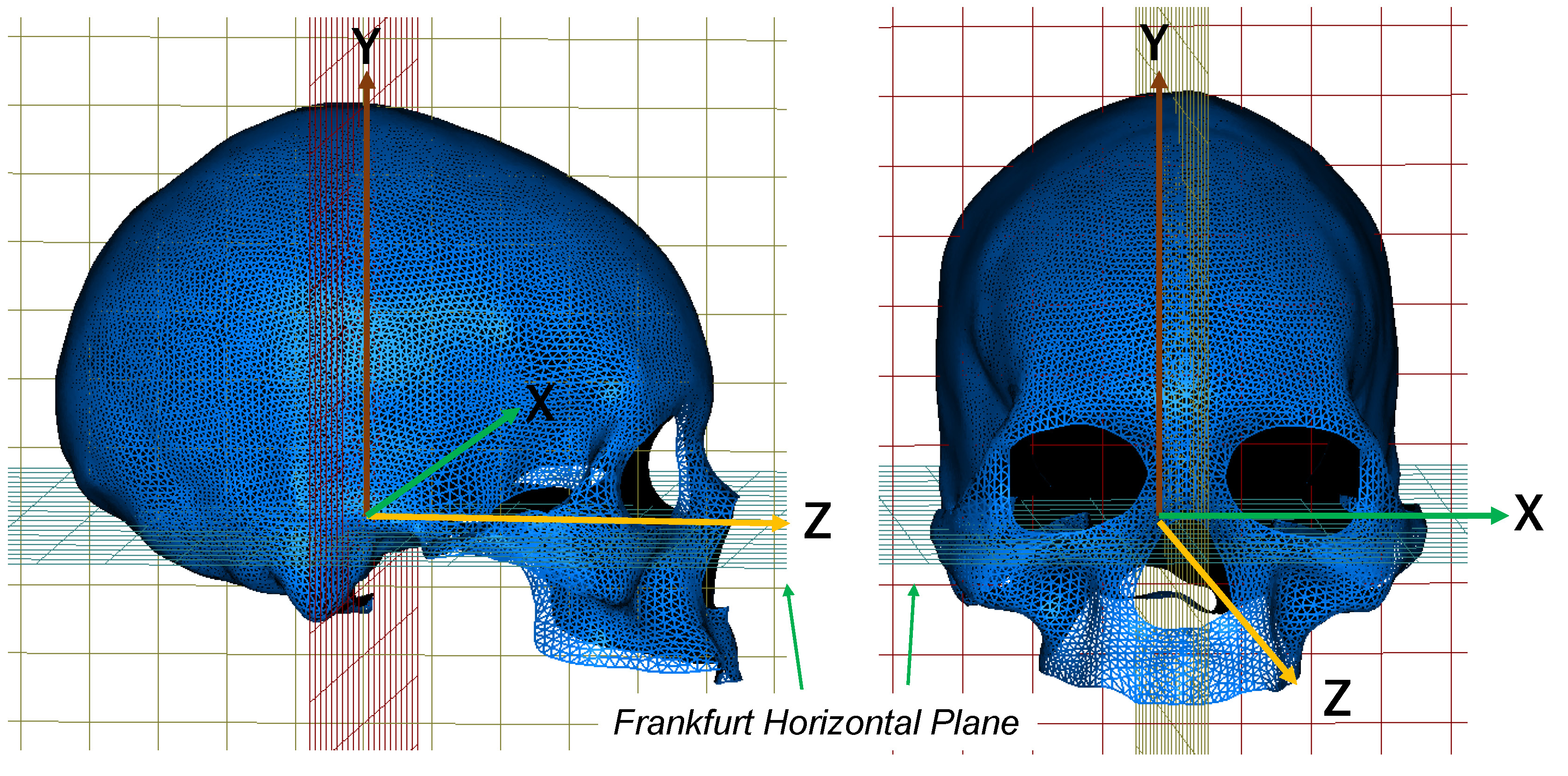

Supplement: Supplementary file 4 — Supplementary Figure S4. [file 41598_2022_15883_MOESM4_ESM.jpg]
