# Supplementary material for: Global patterns of the cranial form of modern human populations described by analysis of a 3D surface homologous model
Source: Sci Rep. 2022 Aug 15;12:13826. doi: 10.1038/s41598-022-15883-3 (PMC9378707; doi:10.1038/s41598-022-15883-3)
Supplement: Supplementary file 19 — Additional Information. [file 41598_2022_15883_MOESM19_ESM.docx]

**Additional Information**

**Separate Supplementary Meta Files**

Table S1. Loading vectors of 14 principal components for 53127 XYZ coordinates of 17709 vertex points on the homologous cranial models.

Table S2. Principal component scores for comparative cranial specimens.

Table S3. The results of ROC analysis applied to loading scores of significant comparative principal components between population sample sets of nine geographical units.

Table S4. The list of cranial specimens used in this study.

Table S5. The list of landmark number and the point.

Table S6. Results of PCA applied to normalized data sets by Centroid size.

Table S7. Principal Component scores based on normalized data sets by Centroid size. (Geographical units: AFR = Africa (Sub-Sahara), AMR = America, EUR = Europe, MEL = Melanesia, MID = Middle East (+North Africa), NEA = Northeast Asia, PMC = Micronesia (included to POL), POL = Polynesia, SAS = South Asia, SEA = Southeast Asia)

Video S1. Sequential morphing images of virtual cranial shape scored in the range between plus or minus 3SD in PC1.

Video S2. Sequential morphing image of virtual cranial shape scored in the range between plus or minus 3SD in PC2.

Video S3. Sequential morphing image of virtual cranial shape scored in the range between plus or minus 3SD in PC4.

Video S4. Sequential morphing image of virtual cranial shape scored in the range between plus or minus 3SD in PC6.

Video S5. Sequential morphing image of virtual cranial shape scored in the range between plus or minus 3SD in PC7.

Video S6. Sequential morphing image of virtual cranial shape scored in the range between plus or minus 3SD in PC3.

Video S7. Sequential morphing image of virtual cranial shape scored in the range between plus or minus 3SD in PC9.

Figure S1. Scatter scoring diagrams of PC2 and PC4, PC6 and PC7, and PC3 and PC9 for the average homologous cranial models of 148 population samples.

Figure S2. Grand-average models of nine geographical cranial series, and the outlines overlapped between the four geographical groups. The scale is given as a green colored sphere of 50 mm in diameter. The bottom

Figure S3. A template mesh model of the cranium used for this study, consisting of 4,485 vertices and 56 landmark points.

Figure S4. The coordinate system applied to the homologous cranial models.
